# Negotiating (Mis-)Recognition in Physical Education: Interactions Between Teachers and Students with Special Educational Needs in the Area of Emotional and Social Development

**DOI:** 10.3390/bs16050793

**Published:** 2026-05-16

**Authors:** Leefke Brunssen, Valerie Kastrup

**Affiliations:** Department of Sports Science, Bielefeld University, 33615 Bielefeld, Germany

**Keywords:** Special Educational Needs (SEN), Emotional and Social Development (ESD), Physical Education (PE), recognition, teacher–student interaction, grounded theory methodology

## Abstract

For students with Special Educational Needs in their Emotional and Social Development (SEN-ESD), school interactions can intensify distrust in adults or foster corrective relational experiences. Physical Education (PE) presents a dual-natured context for this group: while curricula promote social–emotional skill development, students are particularly dependent on sensitive teacher interactions. Yet, no study has examined how recognition, as the prerequisite for inclusion, is negotiated in these teacher–student interactions. This Grounded Theory study reconstructed these negotiation processes and explains them through a Honneth–Prengel recognition framework. Using an iterative design, we conducted and analysed semi-structured interviews with 18 PE teachers and 22 students with SEN-ESD in German regular secondary schools until theoretical saturation. Constant comparative analysis and iterative open and axial coding revealed the dimension of *interactional dignity* (property: level of affirmation; ranging from low ↔ high). Five patterns detail its constitution through three core domains: relational security, fairness and voice, and valuing individual skills. Interactions are strained by perceptual discrepancies, one concerning what counts as just and the other whose reality is recognised. Furthermore, a systemic grading paradox emerged, which may function as institutional misrecognition and may risk double marginalization for students with SEN-ESD, who are assessed on their very area of need in PE. Findings suggest that addressing this requires structural reform beyond teacher practice. Inclusive PE needs resources for individualised pedagogy, teachers who acknowledge individual needs and realities, and systemic reform of assessment practices.

## 1. Introduction

Does Physical Education (PE), where social interactions are constantly negotiated, become a high-risk environment for students with primary needs in this very domain?

Despite the global mandate for inclusive education (United Nations, 2006), students with Special Educational Needs in the area of Emotional and Social Development (hereafter SEN-ESD) continue to experience systemic exclusion (Woodgate et al., 2020). For these students, PE may present a critical opportunity, as curricula across different countries explicitly prioritise social–emotional skill development (e.g., DfE, 2013; KMK & DOSB, 2023; SHAPE America, 2024). Empirical research characterises the gymnasium as a paradoxical arena for students with SEN-ESD (Brunssen & Kastrup, 2025). It can be a domain of success and belonging for students with SEN-ESD, experienced in strong contrast to cognitively oriented subjects (Medcalf et al., 2011), yet general PE research shows it also functions as a site where recognition is allocated as a scarce resource based on performance and normative ideals (Gieß-Stüber & Grimminger-Seidensticker, 2023).

This paper investigates this core contradiction. It argues that for students with SEN-ESD—whose biographies often include fractured trust (Opp, 2017) and underdeveloped social competencies (Myschker & Stein, 2018)—PE becomes a critical arena. Here, the potential for inclusion and student well-being is negotiated and determined in each teacher–student interaction, through a continuous process of (mis-)recognising their dignity and agency. While schools are identified as key institutional and relational settings for promoting children’s social and emotional well-being—where feeling cared for, respected, and valued is foundational (Thomas, 2024)—it remains unclear how this translates for vulnerable students with SEN-ESD in PE. Consequently, no study has examined how students with SEN-ESD and teachers themselves negotiate recognition within the interactions of the PE context.

To address these interconnected gaps, this study focuses on the perceptions of PE teachers and students formally identified in Germany with SEN-ESD (*Förderschwerpunkt Emotionale und Soziale Entwicklung*^1^), who were described by their educators as exhibiting externalising behaviours. We used Grounded Theory Methodology to reconstruct intersubjective recognition processes within teacher–student interactions in PE.

### 1.1. Conceptualizing SEN-ESD and Inclusion

Research categorises Emotional and Behavioural Problems (EBP) into externalising (e.g., aggression, oppositional defiance, hyperactivity) and internalising (e.g., anxiety, social withdrawal, depression) domains (Achenbach et al., 2016; American Psychiatric Association, 2013; Myschker & Stein, 2018). These manifestations can be framed through three lenses, which may coexist (Myschker & Stein, 2018): (1) as mental health disorders defined by diagnostic systems like DSM-5 (American Psychiatric Association, 2013) or ICD-11 (World Health Organization, 2022); (2) as psychosocial problems identified through dimensional taxonomies measuring symptom severity; or (3) as SEN in the area of emotional and social development, requiring educational support. In Germany, diagnosing students for SEN-ESD is governed by state-specific educational legislation and standards of the Standing Conference of the Ministers of Education and Cultural Affairs (KMK & DOSB, 2023).

Students with SEN-ESD represent a growing cohort in German schools, with epidemiological studies indicating high rates of mental health risks, particularly among socio-economically disadvantaged children (Robert Koch-Institut, 2018). Crucially, EBPs often stem from profound adverse experiences. Research links SEN-ESD to histories of neglect, social exclusion, and violence, including early attachment trauma and family dysfunction (Julius et al., 2009; Popp & Methner, 2016). These experiences often lead to a deep-seated feeling of worthlessness and distrust of adults, as children have been disappointed by them in the past (Opp, 2017). When trust is broken through neglect, abuse, or repeated disappointment by caregivers, this manifests as generalised mistrust toward pedagogical institutions and professionals, even when these genuinely demonstrate trustworthiness (Müller, 2017). Such trauma may lead to insecure or disorganised attachment, with internal working models that manifest as school-based mistrust or as conflictual attachment behaviours; these patterns are often dissociated and re-enacted simultaneously as protection and as an unconscious search for corrective relationships (Zimmermann, 2025). In this context, Brisch (2018) emphasises that while children may fear a teacher’s friendliness as a prelude to manipulation or renewed abuse, teachers themselves are among those whom children associate with protection and safety, by virtue of their authority and knowledge. Yet this very authority position means they can also become perpetrators, inflicting further attachment trauma through physical or emotional violence and intensifying feelings of helplessness and powerlessness. Teachers who recognise and act on such signs can serve as “rescuers”, initiating interventions that may remove the child from a harmful environment. Brisch thus calls for teacher training on attachment trauma to enable educators to better understand and support affected students (Brisch, 2018). In sum, students’ behaviour is frequently an expression of underlying pain and shattered trust, making the school environment a critical space for either re-enacting trauma or fostering corrective relational experiences. Pedagogy must therefore foster both trust and healthy mistrust, enabling children to develop sufficient self-trust to discriminate between trustworthy and untrustworthy adults. This can emerge from confirmed self-trust and experiences of recognition within relationships where power asymmetries are explicitly acknowledged (Müller, 2017).

Consequently, scholarship on SEN-ESD consistently advocates shifting from a deficit-oriented to a *resource-oriented perspective* (Guthöhrlein et al., 2025; Prengel, 2019). This shift involves pedagogical practices like solution-focused communication and adopting the role of a guest in the students’ reality (Guthöhrlein et al., 2025) to navigate their often unconsolidated social–emotional competencies (Myschker & Stein, 2018). We ground our understanding of inclusion in Prengel’s concept of *egalitarian difference* (Prengel, 2001), which rejects the binary opposition between equality and diversity: equality without diversity risks undemocratic homogenisation, while diversity without equality entrenches hierarchies (e.g., through stigmatising labels). Accordingly, we define inclusive PE as a pedagogical space where heterogeneity is recognised and valued, where interactions actively counter exclusion and foster embodied, emotional, social and cognitive development alongside a sense of belonging, and where education aims at self-respect and recognition of others (Prengel, 2019). However, Boger’s (2023) trilemmatic theory of inclusion posits that inclusive pedagogical practice is systematically caught between three mutually exclusive goals: *empowerment* (strengthening students’ self-determination and collective voice, often through identity-based claims), *normalisation* (facilitating students’ adaptation to existing norms, e.g., inclusion into mainstream settings), and *deconstruction* (critically questioning the norms and categories that produce difference). Any two of these goals can be pursued together, but doing so necessarily excludes the third, creating structural tensions that teachers must navigate daily.

### 1.2. Recognition Processes in PE

General research in PE demonstrates that recognition is not inherent but is often allocated as a scarce resource based on athletic performance and normative body ideals (Gieß-Stüber & Grimminger-Seidensticker, 2023). This creates systematic recognition deficits for students with low motor skills, among others. The performative, public nature of the gymnasium magnifies these dynamics, where power sources such as sporting ability and social status structure peer interactions and can become public arenas for shame and exclusion (Grimminger, 2012). Furthermore, the implementation of inclusive practices can be complicated, as PE teachers’ socialization can foster preconceived notions and implicit biases about SEN-ESD students, thereby sustaining deficit-oriented interpretations of their behaviour (Brunssen et al., 2025). Sensitive teacher behaviour is directly related to SEN-ESD students’ ability to employ functional emotion regulation strategies in conflictual situations in PE. However, students and teachers in these conflicts can operate from different moral and relational interpretative logics, which can lead to perceptual discrepancies and subsequent conflict cycles (Brunssen & Kastrup, 2025). Recognition processes have been operationalised quantitatively in general PE through the construct of Being Seen. Andresen et al. (2023) identified its core components as opportunities to display competence and receive dialogic feedback from the teacher. Their findings confirm that these factors are positively associated with a student’s overall experience of recognition, quantifying a phenomenon where a significant minority (7.8%) explicitly report not feeling seen. These recognition-related experiences are also motivationally relevant from the perspective of Self-Determination Theory (SDT; Ryan & Deci, 2017): autonomous engagement in PE depends on satisfying the three basic psychological needs of autonomy, competence, and relatedness. In this sense, opportunities to display competence and receive dialogic teacher feedback may support students’ competence and relatedness experiences, with teachers significantly shaping autonomy and competence, while relatedness is co-determined by teacher and peer interactions (Vasconcellos et al., 2020).

The following section presents the analytical framework applied to theoretically elaborate the inductively derived findings in the Discussion (Section 4). Consistent with Grounded Theory methodology, this literature was consulted only after theoretical saturation dignity had proven itself relevant through inductive analysis: to determine the extent to which this category was present in the existing literature (Corbin & Strauss, 2008).

### 1.3. Analytical Framework: Honneth’s Recognition Triad and Its Pedagogical Refinement

Honneth’s (1992/1995) theory of recognition illuminates the moral grammar of social conflict and identity formation. Drawing on Hegel and Mead to affect a materialist turn, he grounds normative claims in the empirical dynamics of social interaction. The theory posits that practical identity development depends on intersubjective recognition, framing societal development as a collective struggle to institutionalise recognition claims. This struggle is structured through three irreducible forms of recognition, each linked to specific identity dimensions, social spheres, and pathologies.

However, recognition is itself deeply ambivalent: institutionalised acts of recognition can be governed by unreflected criteria of recognisability that fix individuals into limiting subject positions, such as ‘the needy’ or ‘the poorly performing’, rather than affirming them unconditionally (Dederich, 2020).

While Honneth’s tripartite model offers insights into moral dimensions of social interaction, its application to childhood and institutional education requires refinement. Scholars in childhood studies argue it does not fully account for children’s specific status as active agents and rights-bearers, not merely passive recipients (Thomas, 2012). To address this and power dynamics in education systems, this research integrates Prengel’s (2019) work, which grounds Honneth’s philosophical framework in empirical educational relationships.

This perspective adopts Honneth’s (1992/1995) *three forms of recognition* with Prengel’s (2019) pedagogical specifications—which reframe *children* as vulnerable yet competent social actors, *bearers of rights*, and unique individuals whose contributions demand esteem—to create a sensitive framework for analysing interactions between PE teachers and children with SEN-ESD in PE. The adapted structure is as follows:

#### 1.3.1. Love: Affective Recognition and Professional Solidarity

This form entails *emotional affirmation* within relationships, grounded in mutual attachment and fostering basic *self-confidence* (Honneth, 1992/1995). For pedagogical contexts, Prengel (2019) reformulates this as *Solidarity with Strangers*—an asymmetrical care relationship characterised by sensitivity and responsiveness, not private affection but professional commitment. It manifests through reliable emotional support and a positive social climate, co-constructed by children whose behaviour signals the solidarity’s success. *Misrecognition* through neglect, psychological violence, or unresponsiveness constitutes professional malpractice that damages self-confidence and increases vulnerability.

#### 1.3.2. Rights: Cognitive Recognition and Equal Freedom

This involves *mutual respect* among rights-bearing persons, enabling *self-respect* through equal rights and participation in collective will-formation (Honneth, 1992/1995). Prengel (2019) specifies this as *Equal Freedom* within pedagogical power relations, operationalised through the core criterion of reversibility: pedagogical action must be conducted in a democratic style that could, in principle, be *reciprocal*. This requires transparent rule systems clarifying participation. Deprivation of this recognition through exclusion, dismissed perspectives, or non-reversible commands constitutes a *misrecognition* that violates social integrity and induces social shame, undermining children’s moral agency (Prengel, 2019).

#### 1.3.3. Solidarity: Evaluative Recognition and Valuing Individual Contributions

This form entails social esteem for individuals’ traits and abilities, enabling positive self-regard and *self-esteem* through valued contributions (Honneth, 1992/1995). Prengel (2019) critiques meritocratic evaluation, proposing a school as a *Caring Community* that values each child’s individual contributions through a resource-oriented perspective. This requires an individual reference norm to appreciate personal progress and potential, separating learning from high-stakes testing. Degradation or insult constitutes *misrecognition*, which Prengel identifies as an *adultist and ableist practice* that reinforces deficit-oriented labelling.

This synthesised framework is employed to analyse recognition processes from students’ and teachers’ view in interactions in inclusive PE from both perspectives.

### 1.4. The Present Study

While research establishes that recognition in PE is performatively allocated (Gieß-Stüber & Grimminger-Seidensticker, 2023) and scholarship asks for resource-oriented pedagogy for students with SEN-ESD (Guthöhrlein et al., 2025; Prengel, 2019), these two strands of literature have developed in parallel. The particular dependency of students with SEN-ESD on sensitive interactions in this socially demanding context is acknowledged (Brunssen & Kastrup, 2025), yet no study has empirically investigated the interactive mechanisms linking them—specifically, how teachers and students mutually perceive and negotiate (mis-)recognition in inclusive PE.

We conducted a broader Grounded Theory study (Corbin & Strauss, 2008; Breuer et al., 2018) with the open aim of understanding which themes and situations are relevant for students with SEN-ESD and their PE teachers. The Honneth–Prengel recognition framework was not known to the researcher prior to or during the coding phase; it was identified only after theoretical saturation. Once engaged, this framework may have implicitly deepened the interpretation of findings and explicitly has shaped the theoretical elaboration and organisation of findings in the Discussion. Through inductive data collection and analysis, interactional dignity, which we later theoretically conceptualised as (mis-)recognition, emerged as a category shaping teacher–student interactions. The present manuscript reports on this category, examining how it is negotiated from the perspectives of both groups.

Research question:

*How do students with Special Educational Needs (SEN) in their Emotional and Social Development (ESD) and Physical Education (PE) teachers perceive (mis-)recognition in Teacher–Student Interactions in inclusive PE settings?*

## 2. Materials and Methods

This study employed a *Grounded Theory approach* (Corbin & Strauss, 2008), supplemented by methods from Reflexive Grounded Theory (Breuer et al., 2018). Corbin and Strauss’s (2008) methods guided coding, constant comparison, and category development. Breuer et al.’s (2018) methods were added to manage the researcher’s prior professional knowledge as a former PE teacher with direct experience teaching students with SEN-ESD. To make this knowledge epistemologically productive, we used methods of pre-concept explication at project outset and continuous self-reflexive memoing throughout the coding process (Breuer et al., 2018; Corbin & Strauss, 2008).

Using constant comparative analysis, we inductively explored themes in inclusive PE for students with SEN-ESD. Qualitative, semi-structured interviews were conducted to access implicit processes. Between November 2023 and April 2025, 18 PE teachers (coded T1–T18, ‘T’ = teacher) and 22 students (coded S1–S22, ‘S’ = student) with SEN-ESD (ages 10–16) from general education secondary schools in three German states were interviewed. No pre-existing relationships existed between teacher and student participants. Student *inclusion criteria* were: a formal SEN-ESD diagnosis established by a special educator in accordance with German state-specific legislation and the standards of the Standing Conference of the Ministers of Education and Cultural Affairs (KMK & DOSB, 2023); they were described by their responsible (special) educator as exhibiting externalising behaviours, no standardised behavioural assessment was conducted; enrolment in a general secondary school; and no autism spectrum disorder diagnosis. Teacher inclusion criteria were: qualification as a general (not special) PE teacher; and active teaching in PE of at least one student meeting the student inclusion criteria at the time of the interview.

Participants were *recruited* via school principals; some PE teachers were additionally contacted directly about their willingness to participate. Student eligibility was confirmed through a multi-source process: legal guardians received and signed informed consent documents explicitly stating that the study involved solely students with a formal SEN-ESD diagnosis; eligibility was further confirmed by the responsible special educator or class teacher, who are required in Germany to maintain individual educational support plans for students with SEN; and all school principals were informed about and approved participation of the specific student or teacher.

All *interviews* were conducted individually. Interview duration ranged from 12 to 43 min for students and from 20 to 106 min for teachers, reflecting the open-ended, narrative nature of the protocol and the depth of individual accounts. Following a description of their most recent PE session, all participants recounted a memorable PE situation, as well as positive and challenging situations in PE, with follow-up questions probing inductively relevant details. Teachers focused on a situation involving a current student with SEN-ESD. Interviews were audio-recorded, transcribed verbatim, and anonymised. Using MAXQDA 24, constant comparison was applied across interviews and memos. Quotes are identified by participant code (e.g., S1 for Student 1) and a position number (Pos.), for each speaker turn. The analysis was conducted by a researcher trained in Grounded Theory methodology.

**Figure 1:**
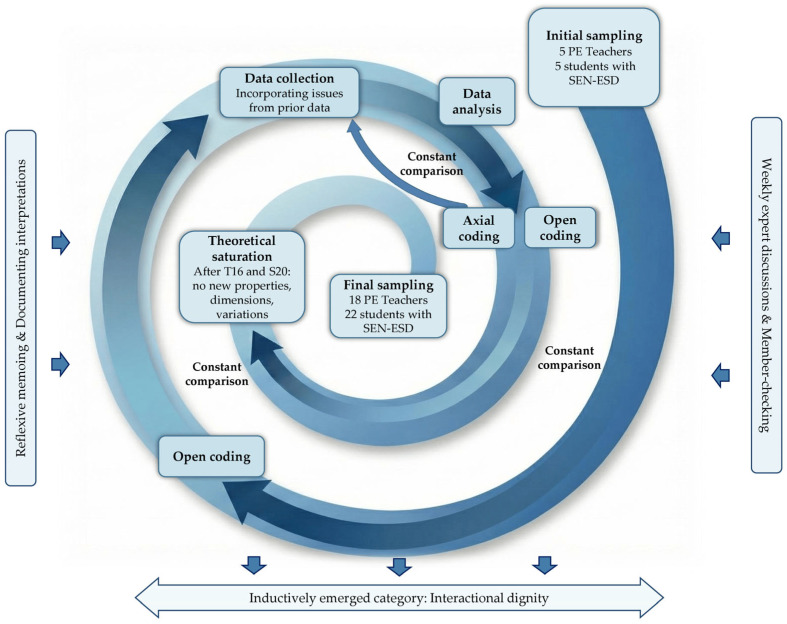
Grounded Theory research process with the emerging category ‘Interactional Dignity’.

Guided by theoretical sampling, the *analytical process* proceeded iteratively. An initial sample of 10 interviews was openly coded line-by-line to fracture data and identify provisional concepts. To maximise variation, the interview displaying the strongest contrasts was selected for subsequent open coding. Following open coding of 4–5 interviews per group, the analysis progressed iteratively between open and axial coding (Figure 1). Axial coding grouped and related concepts, dimensionalising their properties to develop a coding paradigm for understanding the data from both groups. Initially, a separate axial coding paradigm was developed for each group. As the level of abstraction increased through constant comparison, it became evident that teachers and students addressed many of the same themes and situations, but perceived and articulated them in divergent ways. This data-driven convergence justified merging both group-specific paradigms into a single integrated coding framework. Where certain sub-categories and patterns remained exclusively relevant from one group’s perspective, this is discussed at the respective point in the Results (Section 3).

Within the broader *Grounded Theory of the larger project*, the present manuscript reports on one category, *interactional dignity*, which was dimensionalised along the property *Level of Affirmation*, ranging from *low* to *high*. In the broader theory, the *central phenomenon* is perceptual discrepancies, comprising five variations, each explaining why and how (inter-)actions can be perceived differently by different participants. These divergent perceptions arise from diverging underlying interpretive patterns: perceptual discrepancy denotes the phenomenon of divergent interpretation itself, whereas interpretive patterns refer to the enduring cognitive schemas that systematically generate these divergences. From the data constituting the category of interactional dignity, two of these variations emerged as particularly salient: a *justice-related perceptual discrepancy* concerning what counts as fair, and an *epistemic discrepancy* concerning whose subjective reality is recognised. While these variations can co-occur and overlap across categories and patterns, both are most fully manifested in Pattern 3, where fairness and voice are the explicit subject of interactional negotiation.

*Theoretical sampling* focused on sampling categories and variations rather than specific groups of individuals. New interviews were conducted iteratively, incorporating issues from prior data. Theoretical saturation was assessed at the level of the overall research project, of which this manuscript forms one part (see also Brunssen & Kastrup, 2025). Data collection continued until all categories were saturated (February 2025). Consistent with Corbin and Strauss (2008), saturation was reached when all categories were well-defined and developed in terms of their properties and dimensions, with sufficient variation built in to show the range to which each applies. All five patterns of interactional dignity were identifiable within the first ten interviews; subsequent theoretical sampling focused on developing their properties and dimensions in depth. Different patterns were identified and subsequently synthesised through analytical condensation. No new properties or dimensional variations emerged after the 16th teacher and 20th student interview; two further interviews per group confirmed robustness.

Consistent with *Reflexive Grounded Theory* (Breuer et al., 2018), the researcher’s prior professional knowledge as described above was managed throughout the coding process; the Honneth–Prengel recognition framework was not known to the researcher at this stage. Following theoretical saturation, the researcher entered what Corbin and Strauss (2008) describe as the writing stage, in which “the literature can be used to confirm findings […]. Bringing the literature into the writing […] allows for extending, validating, and refining knowledge in the field” (p. 38). At this point, a member of the weekly expert group—following the established protocol in which the researcher presents data while remaining silent to enable independent interpretation—identified resonances with Honneth’s recognition spheres; this was documented in an analytic memo and pursued in subsequent expert discussions before the framework was formally engaged. As engaging with literature may subconsciously influence a researcher, the Honneth–Prengel framework may have implicitly influenced the interpretation of these findings (Corbin & Strauss, 2008); intentionally, it did shape the theoretical organisation of the Discussion. The codebook, interview protocol, and representative analytical memos documenting this sequence are available from the corresponding author upon request.

*Validation* was strengthened through weekly interdisciplinary discussions with four other Grounded Theory experts (Corbin & Strauss, 2008). Theoretical sensitivity was maintained through reflexive memoing, documenting researcher interpretations and decision-making processes (Breuer et al., 2018). Formal member-checking with students was ethically precluded due to their age and vulnerability; however, findings were presented to and discussed with seven practicing PE teachers in total, who reviewed the identified patterns for plausibility and confirmed that they resonated with their professional experience.

## 3. Results

**Figure 2:**
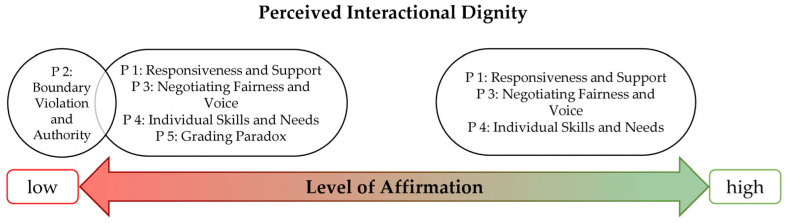
Mapping the five emergent Patterns onto the Dimension ‘Interactional Dignity’.

The analysis of the category *interactional dignity* was structured along the property *Level of Affirmation*, ranging from low affirmation (misrecognition) to high affirmation (recognition). As illustrated in Figure 2, no patterns were perceived in an ambivalent range in this category, but the five patterns were located either in the low-affirmation range or in the high-affirmation range. For Patterns 1, 3, and 4, data were reconstructed in both ranges (low and high). Patterns 2 and 5, which emerged solely from the student perspective, were mapped only in the low-affirmation range; notably, Pattern 2 is mapped furthest toward the low-affirmation pole, reflecting very low or no affirmation due to the absence of perceived negotiation in these interactions. Another notable finding was the *consistent positioning of individuals* within the same range *across patterns*. The primary exception was students recounting experiences with different teachers; such differentiated positioning was not found for teachers.

### 3.1. Pattern 1: Responsiveness and Support

*High: Relational Security*

Teacher: “I find a small conversation […], a ‘How are you today?’ […] always going into these reflections, is very important.”

(T9, Pos. 56)

Student: “He showed immediate understanding. Said: ‘Okay, I understand. If something is wrong, please let me know so I know what’s going on with you.’”

(S21, Pos. 42)

*Low: Emotional Abandonment*

Teacher: “[I find her] incredibly annoying. I really have to bite my tongue sometimes to not get rude with her […] simply nerve-wracking”

(T2, Pos. 37)

Student: “He showed no consideration for me. He sometimes said ‘Get out NOW’ or he just called my mother, didn’t try to talk with me.”

(S21, Pos. 38)

The *high affirmation pole* demonstrates how teachers’ check-ins and calming strategies can support relational security. Teachers describe establishing predictable support through simple check-ins like asking “How are you today?” (T9, Pos. 56) and approaching conflicts with empathy and structured cooling-off periods. Students experience this as reliable support from teachers who intervene “immediately when something happens” (S16, Pos. 138) during peer conflicts and maintain constant availability. This consistent responsiveness can foster mutual trust. Students describe feeling more willing to signal their individual needs in these contexts, which “varies from student to student.” (T18, Pos. 29). Teachers’ empathy and validation of students’ subjective experiences “I will pay more attention to the observation you are making” (T5, Pos. 16) is associated with more secure relational dynamics in participants’ accounts

Conversely, the *low affirmation pole* reveals how emotional neglect damages students’ sense of safety. Students reported teachers sometimes failed emotional detection by remaining passive during peer conflicts “the teacher just watches and does nothing about it”, leaving them feeling abandoned and uncertain (S20, Pos. 79). In some cases, this neglect escalated to active dismissal, such as when teachers minimised students’ perceived physical harassment as mere playfulness “just for fun and I should see it as fun” (S18, Pos. 112) or expressed overt disinterest in students’ perspectives. The resulting relational damage can create profound distrust, with students convinced that seeking help is pointless (S15, Pos. 88). This pattern is reinforced through punitive reactions like immediate exclusion where a teacher “doesn’t really clarify things” (S10, Pos. 71) in conflict mediation, which can undermine students’ sense of security and worth within the classroom community.

### 3.2. Pattern 2: Boundary Violation and Authority

Student: “And then he [the PE teacher] freaked out, screamed at everyone […] He didn’t give any warning, but sent us to get changed right away.”

(S19, Pos. 11)

This pattern, documented from students’ perspective and only on the *low affirmation pole* of the dimension, reveals how abusive authority fundamentally undermines interactional dignity by destroying relational safety. Students experienced immediate, escalated responses to minor misbehaviour and arbitrary punishment without warning or explanation. One student describes “In PE, um, he shouts without reason. He tries to intimiDATE the children.” (S5, Pos. 17). This authoritarian approach was characterised by a complete disregard for student perspectives, with teachers focusing solely on their own agenda rather than engaging in dialogue. In one case, these violations extended to physical intrusions, including a student being pulled by their clothing—a direct breach of bodily integrity that destroyed the student’s sense of physical safety. Others felt publicly humiliated when teachers “started screaming at me in front of the whole class” while assigning blame without investigation (S20, Pos. 170). One student contrasted this with a desire for teachers who would reduce power distance and “Not just brutally push through the lesson, but do it as if he also enJOYS it” (S19, Pos. 62). Pattern 2 is documented exclusively from students’ accounts and is mapped furthest toward the low-affirmation pole; as students perceived dialogue was foreclosed, no negotiation process occurred. The absence of teacher accounts permits three interpretations. First, it may reflect a perceptual discrepancy: low-affirmation teacher accounts in Pattern 3, where teachers explicitly endorse authoritarian approaches, may partly constitute the teacher perspective on interactions students reconstruct as boundary violations. Second, teachers may not have reported such conduct in interviews, for instance because of social desirability. Third, it may reflect a sampling limitation: no teacher who engaged in such conduct was interviewed. Because these alternatives cannot be distinguished on the basis of available data, no additional discrepancy type is established from this pattern.

### 3.3. Pattern 3: Negotiating Fairness and Voice

*High: Co-constructed Fairness*

Teacher Quote: “I want to be taken seriously and that’s how I definitely treat you too.”

(T3, Pos. 25)

Student Quote: “Now you have these two options […] then I usually choose the option that has more advantages for me.”

(S21, Pos. 123)

*Low: Authoritarian Control*

Teacher Quote: “A democratic educational style would not be appropriate for them […] Rather, a militaristic style”

(T2, Pos. 88)

Student Quote: “It’s impossible to discuss with him [PE teacher]. […] because he has a higher position than us students, he can do whatever he wants. He doesn’t let you explain at all. […] you have no chance to express yourself about it.”

(S19, Pos. 35)

In Pattern 3, which captures the most statements in the dataset, the *high affirmation pole* demonstrates how transparent differentiation and consistent rule enforcement create interactional dignity. Teachers practice differentiated transparency by explaining to the class why students with SEN-ESD receive adjusted consequences, for instance, making clear “why the student is freaking out again and why he is allowed to come back” (T4, Pos. 61), navigating tensions between formal equality and substantive justice. Students, however, link fair treatment to procedural consistency, valuing a teacher who “steps in and enforces them properly. […] He takes the rules very seriously” (S19, Pos. 135). Interactional dignity is additionally fostered by respecting student autonomy via participatory engagement. Teachers do this by providing meaningful choices, creating space for autonomous conflict resolution by holding back, and practicing impartiality that resist premature assumptions, as one teacher notes, “with students who are conspicuous, I think the most fatal thing is to always assume that the stress originated from him or her” (T5, Pos. 24). Students experience this through democratic practices like majority voting and reflective dialogue. Thus, interactional dignity is constituted through both consistent fairness and authentic recognition of agency.

Conversely, the *low affirmation pole* reveals how arbitrary treatment and stigmatization can undermine perceived dignity. Students experience inconsistent rule enforcement, reporting “I was suspended […] and she was allowed to stay” due to being perceived as “that kind of person who often starts conflicts” (S20, Pos. 107). This is compounded by stigmatization patterns, where students report feeling pre-judged by teachers based on behavioural labels: “[they] always have huge problems admitting that they were hit or that they lost, and they simply cannot deal with frustration” (T15, Pos. 17). Furthermore, authoritarian control denies moral agency, such as explicitly rejecting democratic approaches. Students experience this through systematic exclusion from explanatory processes, having “no chance to express yourself” (S19, Pos. 35), which manifests as one-sided game modifications, immediate dismissal of student suggestions: “when you can make a suggestion, then Mr. Martens says ‘No’” (S14, Pos. 111), and punishment for questioning unfair treatment. From the students’ perspective, we reconstructed these practices can signal that their views are less important and their self-determination unrecognised, constituting a recurring violation of interactional dignity in our data.

Underlying these contrasting experiences of fairness and voice, two intertwined *perceptual discrepancies* became evident in the data: one concerning *what counts as fair*, the other concerning *whose reality is recognised*. Both interpretive logics were reconstructed inductively through constant comparative analysis of cases in which fairness, voice, and the recognition of perspectives were contested; they represent socially shared interpretive patterns identified across participants on both sides and consistently generate predictable interpretations in new situations (Corbin & Strauss, 2008). As detailed in the Methods section, while these logics have partial resonance across patterns, both are most fully manifested in Pattern 3.

For the *first discrepancy*, an underlying *justice-related interpretative logic* was reconstructed: teachers and students alike held two competing justice logics. Some participants, both teachers and students, insisted on procedural equality: the same rules and consequences for everyone. Others, again on both sides, argued for substantive justice, where needs-based differentiation is fairer. Teachers who followed the latter logic consciously allowed more leeway, such as “letting things slide twice as often for Lukas” (T18, Pos. 39); students who held this perspective wished for “a bit more understanding for people with, for example, intellectual disabilities” (S21, Pos. 146) rather than rigid equality. As a result, needs-based differential treatment of rule violations—intended as substantive justice—clashed with the demand for procedural equality, leaving both sides (teachers and students alike) perceiving the other’s logic as unfair.

For the *second discrepancy*, concerning whose reality counts as valid, an underlying *epistemic interpretative logic* came into view. This logic is not merely about power, it reflects a deeper conflict over whose version of reality is granted legitimacy. Some teachers operated from an objectifying, external framework, while students spoke from their subjective experience. One student articulated the resulting asymmetry directly: “because he has a higher position than us students, he can do whatever he wants” (S19, Pos. 35). Instead of engaging, teachers might dismiss the student’s reality by marking it as “this feeling of being treated unfairly is, well, extremely, extremely subjective” (T10, Pos. 42), responding “That’s not correct how you see things” (T10, Pos. 21), thereby devaluing the student’s perspective as irrational rather than negotiating it. In contrast, other teachers engaged the student’s subjective reality, recognising that what mattered to students was not perfect implementation but the genuine effort to take their perspective seriously: “one should at least try, that’s already enough for me” (S21, Pos. 86). These teachers worked toward clarifying differing viewpoints together, ensuring “that they feel taken seriously” (T5, Pos. 16).

### 3.4. Pattern 4: Individual Skills and Needs

*High: Valuing Unique Contributions and Abilities*

Teacher Quote: “And then I can talk with him […]. I have to say, his self-reflection is enormous.”

(T18, Pos. 25)

Student Quote: “For instance, when you’re playing well. It’s nice to get positive feedback from the teacher or from friends in general.”

(S1, Pos. 30)

*Low* *: Overlooked Efforts and Standardised Tasks*

Student Quote: “This is too boring for me. […] We practice all these grips and how to fall. I already know all of this.”

(S14, Pos. 53)

The *high affirmation pole* demonstrates how affirming students’ individual strengths and needs fosters social dignity by valuing their participation. Teachers provide specific, differentiated recognition of both personal attributes and motor skills, such as praising a student’s exceptional self-reflection or analytically describing a student’s athletic profile with precise strengths “she’s good at dribbling and in one-on-one situations” and areas for growth (T12, Pos. 10). Educators emphasise that “differentiation is very important”, noting that “it doesn’t have to be that everyone achieves the same thing or in the same way” and that with such an approach “she also feels comfortable” (T16, Pos. 16), ensuring every student can experience success according to their capabilities and rejecting a one-size-fits-all approach. Students experience this recognition as meaningful positive feedback for their performance and appreciate competent, tailored teacher support. Furthermore, most students expressed strong motivation for physical activity. They described PE—often in contrast to other subjects—as a domain where they see themselves as competent athletes and feel confident in their motor skills.

In contrast, students describe situations where their individual skills and learning needs are unaddressed. They report frustration with standardised tasks “we all had to do the same thing “, such as mandatory gymnastics sequences where students with varying prior experience all faced the same requirements (S11, Pos. 49). Some find activities repetitive when they already master the skills being taught “I already know all of that” (S14, Pos. 53), while others feel unsupported during complex tasks like choreographing dances due to insufficient guidance. Students also mention situations where teachers focus primarily on social play rather than technical skill development or provide critical feedback without demonstrating techniques. In some cases, students perceive that teachers pay more attention to high-performing peers. From the students’ perspective, we reconstructed that in these situations they feel their abilities and learning needs are overlooked, diminishing their sense of being valued members of the learning community.

### 3.5. Pattern 5: Grading Paradox

Student Quote: “Our previous [teacher] didn’t pay attention to handicaps or anything. For example, with my emotions, he gave me bad grades for that.”

(S21, Pos. 130)

Students’ accounts reveal a perceived tension between standardised grading practices and their individual learning needs. Students report their grades in PE are determined not only by motor skills but predominantly by behavioural and social–emotional criteria like “good behaviour,” “teamwork,” and “how we participate” (S7, Pos. 43; S8, Pos. 56–61). However, students repeatedly describe situations where these assessment practices feel inconsistent or fail to account for their individual circumstances. Some report feeling penalised for social difficulties despite their efforts to participate, while others perceive an inconsistent application of standards, such as inactive peers, who “were just sitting around in PE and talking”, receiving comparable grades to participating students (S18, Pos. 80). In one case, a student received lower grades specifically for behaviours related to her diagnosed needs, without apparent accommodation: “he didn’t pay attention to handicaps or such. Like for example with my emotions, he gave me bad grades for that” (S21, Pos. 130). Most students express confusion about the connection between their behaviour and their academic assessment. From the students’ perspective, we reconstructed that these grading practices can make them feel that their individual efforts and challenges are not adequately recognised within the evaluation system. This creates situations where the assessment process itself becomes a source of perceived injustice rather than a fair measure of their capabilities and development.

### 3.6. Systemic Barriers to Dignity

Beyond the dyadic teacher–student interactions, teachers’ accounts highlight how systemic constraints limit their capacity to foster interactional dignity for all students. A primary barrier identified is a *scarcity of resources*, creating situations of systemic misrecognition. Teachers describe being structurally unable to meet diverse needs, feeling torn between supporting students with SEN-ESD and their duty to the rest of the class, ultimately leading to instruction tailored to the “average” student (T2, Pos. 90; T1, Pos. 47). This is compounded by a reported *lack of specialised training* especially for students with SEN-ESD, leaving teachers feeling overwhelmed and poorly equipped without the necessary support staff, such as school assistants, special needs educators and psychologists (T10, Pos. 30). Consequently, in their eyes, the lack of institutional support pits students’ rights against each other, making it very *difficult*, under current conditions, to fully address any individual student’s needs.

### 3.7. Peer Dynamics as Barriers to Dignity

Student accounts reveal that peer interactions themselves can undermine or support their perceived social dignity within the learning community. For many, a foundational sense of security is connected to *reliable friendships* and *equitable treatment* within their *peer group*. To many, their sense of dignity is undermined by violations of fair play, where cheating, persistent arguing over rules, and dishonest conduct—such as a player refusing to admit they were tagged—reduce their enjoyment and sense of justice in games (S9, Pos. 133; S8, Pos. 101). Beyond rule-breaking, many students describe a climate of peer conflict characterised by frequent insults, verbal aggression, and physical fights. However, many report that they do not find this climate burdensome, but rather perceive it as a normal part of school life.

## 4. Discussion

This Grounded Theory study is, to our knowledge, the first study to examine the dynamics of *interactional dignity* in inclusive Physical Education (PE) from the perspectives of students with Special Educational Needs in the area of Emotional and Social Development (SEN-ESD) and their PE teachers. Four *main results* emerged:

*First*, we identify *three* interconnected *domains of (mis-)recognition*: (a) *emotional support and safety* in patterns 1 and 2, contrasting attunement with boundary violations; (b) *fairness and voice* in pattern 3, where autonomous engagement conflicts with stigmatization; and (c) *individual valuation* in patterns 4 and 5, where recognising unique strengths fosters self-worth, while standardised approaches penalise developmental needs.

*Second*, our analysis reveals two *perceptual discrepancies*: one concerning what counts as fair, the other concerning whose reality is recognised.

*Third*, *contextual layers* mediate these interactions in PE: teachers experience systemic barriers, a lack of time and wish for SEN-ESD related training, often leading to one-size-fits-all instruction, while students’ sense of social safety and dignity is directly shaped by peer dynamics of (un)fair play.

*Fourth*, pattern 5 reveals a systemic grading paradox that institutionalises misrecognition, grading students on their very area of need and producing a double marginalization.

For the *first main result*, our first domain *emotional support and safety* (Patterns 1 & 2), can be theorised as manifestations of Honneth’s (1992/1995) *Love* or *Affective Care* sphere. The first pattern *Responsiveness and Support* shows that students experience recognition as a form of what Prengel (2019) conceptualises as *Professional Solidarity*—a reliable, affective affirmation of their needs. This is exemplified by teacher practices such as proactive check-ins and empathetic perspective-taking, which supported the students’ sense of a secure relational base (Honneth, 1992/1995). Empirical research on students with SEN-ESD in PE confirms that such sensitive teacher behaviour is directly related to students’ ability to employ functional emotion regulation strategies in conflictual situations (Brunssen & Kastrup, 2025). Conversely, the *misrecognition* of this pattern through emotional neglect in student–teacher dialogues or passive observation during peer conflicts may damage this trust. From the students’ perspective Professional Solidarity (Prengel, 2019) extends to peer dynamics: while reliable friendships supported students’ sense of belonging, they described a climate of peer conflict characterised by aggression, which many did not perceive burdensome. Several explanations may account for this normalization. First, for students with SEN-ESD, whose biographies frequently include fractured trust and persistent exposure to aggression (Opp, 2017; Müller, 2017), such normalization may reflect their biographical experiences. Second, the frequency of peer conflict perceived in PE may itself contribute to normalization. Third, this normalization aligns with findings from our broader Grounded Theory project, in which some students interpret peer aggression as a morally coherent reciprocal response (Brunssen & Kastrup, 2025). Consequently, the teachers’ role in enacting professional solidarity extends beyond direct interactions to include the active cultivation of a supportive peer environment. *Pattern 2, Boundary Violation and Authority*, documented only from the student perspective, reveals a more severe disruption of the *affective sphere*, where a professional teacher role is lost and graduated de-escalation does not occur in our data. Students perceive these interactions as deeply damaging, with explosive anger, public shaming, or physical intrusion constituting what this study interprets, drawing on Honneth (1992/1995), a disruption of the holding function essential for care relationships. This can be especially problematic for students with SEN-ESD, who frequently have biographies marked by experiences of neglect, disrespect, and violence (Julius et al., 2009; Popp & Methner, 2016; Zimmermann, 2025) and whose failed trust relationships can lead to deep-seated distrust of adults in general (Müller, 2017). For students with histories of adverse relationships, such boundary violations may, drawing on the theoretical framework of Brisch (2018) and Opp (2017), function as re-traumatizing experiences that confirm negative self-perceptions and compound distrust of adults in institutional settings. While the data do not directly measure this process, students’ accounts of profound distrust and protective withdrawal are consistent with such theoretical expectations. Thus, the professional holding function requires reflective capacity to prevent pedagogical interactions from exacerbating a traumatic process (Zimmermann, 2025). The gymnasium’s performative, public nature may even intensify this, as power dynamics can create an arena for public shame and exclusion (Grimminger, 2012). In our data, such violations often lead to defensive self-exclusion, representing a protective withdrawal from the very interactions essential for their social–emotional development. Based on the characteristic tendency of students with internalising Emotional and Behavioural Problems (EBP) toward passivity and social withdrawal (Achenbach et al., 2016), boundary violations of this kind may be less likely to occur for these students; their dignity violations may instead take the form of overlooking, where distress expressed through withdrawal rather than disruption goes undetected by teachers.

The second domain, fairness and voice, emerging from our *first main result* as Pattern 3, manifests what Honneth (1992/1995) identifies as the *recognition* sphere of *Rights* or *Cognitive Respect*. In the school context, Prengel (2019) specifies this as the realization of Equal Freedom for children. Recognition establishes students as rights-bearing persons in two ways: First, through equal rights (Honneth, 1992/1995), which students perceived as manifest in transparent and consistent rule application especially in games, which created a just order. Second, through participatory practices, such as being offered meaningful choices and included in democratic processes. These practices embody the solution-focused communication culture that initiates a common dialogue, which are especially important for students with SEN-ESD (Guthöhrlein et al., 2025). Teachers’ transparent explanations and respect for autonomy thereby fulfil the right to participation (United Nations, 2006), acknowledge students’ moral agency, and meet Prengel’s (2019) criterion of reversibility. Conversely, the *misrecognition* of these patterns undermines this status. Misrecognition manifests as arbitrary treatment and stigmatizing generalizations, which induce social shame, and as authoritarian control that excludes students from explanatory processes. This approach imposes unquestionable commands, exemplified by a teacher who explicitly rejected democratic engagement for all students with SEN-ESD. This rejection of a reciprocal manner violates Prengel’s (2019) criterion of reversibility, which constitutes a misrecognising interaction that denies students’ moral agency. Such preconceived notions, often rooted in PE teacher socialization (Brunssen et al., 2025), can lead to the discriminatory labelling Prengel (2019) condemns, which pathologizes students, reduces them to a diagnostic category, and fails to recognise their unique worth.

Finally, the third domain in our *first main result* of *individual valuation* (Patterns 4 and 5) corresponds to the sphere of Social Esteem or Solidarity (Honneth, 1992/1995). When teachers provide differentiated feedback, such as praising a student’s “enormous” self-reflection (T18, Pos. 25) or precisely analysing their athletic profile (T12, Pos. 10), students reported feeling empowered as their unique contributions were acknowledged. This connection aligns with Honneth’s (1992/1995) developmental potential of recognition: such experiences may support both, individualisation by affirming students’ distinct characteristics, and *equalization*, by securing their standing within the classroom community—an outcome that can be particularly important for this vulnerable student group. This practice resonates with findings in PE research, which identify being able to display skills and receiving tailored feedback from the teacher as core components of students’ experience of Being Seen (Andresen et al., 2023). Critically, our data show that such feedback can move beyond a meritocratic logic, as criticised by Prengel (2019), towards a resource-oriented perspective that is also advocated by Guthöhrlein et al. (2025), which values a child’s individual physical or social skills and allows students to experience this as a validation of their unique abilities within the class as a community of value.

Our *second main result* reveals that two intertwined *perceptual discrepancies*, which empirically mainly derived from Pattern 3, can impede affirming recognition in teacher–student interactions: one concerning what counts as fair, the other concerning whose reality is recognised. This finding aligns with (Brunssen & Kastrup, 2025), who identified perceptual discrepancies rooted in moral and relational interpretative logics, especially in conflictual teacher–student relationships in PE with SEN-ESD students. Our results extend this by identifying at least two additional underlying interpretative logics that give rise to such perceptual discrepancies. *First*, the underlying *justice-related interpretative logic* emerged as a clash between procedural equality (same rules for all) and substantive justice (needs-based treatment of rule violations), held by both teachers and students alike. The public, performative nature of the gymnasium (Grimminger, 2012) likely intensifies this clash, because differential rule enforcement is visible to peers and can be experienced as arbitrary favouritism or as neglect. One teacher attempted to navigate this by practising radical transparency, explaining to peers: “we let things slide twice as often for Lukas” (T18, Pos. 39). While this aims to reconcile equality and individual needs, it illustrates the tension Boger (2023) described in the trilemma of inclusion: it pursues empowerment and normalisation, but at the cost of deconstruction—publicly fixing a difference that may stigmatise students (Dederich, 2020). From our data, students’ accounts confirm that such transparency, however well-intentioned, is not universally experienced as affirming. Nevertheless, even transparent rule-based solutions *cannot fully resolve the justice discrepancy*; both sides continue to perceive the other’s logic as unfair. *Second*, the underlying *epistemic interpretative logic* concerns whether teachers validate students’ subjective experiences as legitimate or dismiss them as merely subjective. This directly relates to Andresen et al.’s (2023) construct of Being Seen in PE: dialogic feedback and feeling seen require that teachers take students’ perspectives seriously. When a teacher labels a student’s feeling as “extremely, extremely subjective” (T10, Pos. 42) or responds “That’s not correct how you see things” (T10, Pos. 21), recognition is foreclosed. Such epistemic dismissal may be particularly damaging for students with SEN-ESD, many of whom have histories of having their reality denied in adverse relationships (Müller, 2017; Brisch, 2018), thereby re-enacting rather than correcting mistrust. In contrast, other teachers engaged the student’s reality as equally valid. One teacher worked toward clarifying differing viewpoints together, adopting the role of a “guest” in the student’s construction of reality (Guthöhrlein et al., 2025, p. 458), prioritising that the students “feel taken seriously” (T5, Pos. 16). This constructive stance aligns with resource-oriented pedagogy and may, in Honneth’s (1992/1995) terms, resonate with both the rights sphere (voice, equal freedom) and the love sphere (affective validation). However, even with such effort, the trilemma persists: fully validating a student’s subjective reality may conflict with institutional demands for objective accountability.

Our *third main result* shifts the lens from the pedagogical trilemma to a systemic root: it reveals that this imperative is constrained by *institutional failures*. Teachers report that a scarcity of resources and specialised training for supporting students with SEN-ESD renders them unable to meet complex demands. This systemic scarcity not only pits students’ rights against each other but prevents the realization of the school as a Caring Community (Prengel, 2019), where a resource-oriented perspective values each child’s individual contributions. Consequently, cultivating a classroom environment where all students, especially those vulnerable to peer conflict, can experience full interactional dignity and self-confidence (Honneth, 1992/1995) becomes *severely constrained*, posing a structural challenge that individual teachers alone cannot resolve within current conditions. The *students* in our study predominantly expressed strong *motivation for physical activity* and largely perceived their *motor skills* as competent. PE emerged as a subject where they could achieve individual recognition and feel successful, which some of them contrasted with more cognitively oriented subjects. This aligns with findings that PE can serve as a unique domain of success for students with SEN in the area of Social, Emotional, and Behavioural Difficulties (Medcalf et al., 2011). From a Self-Determination Theory (SDT) perspective, students’ strong motivation and sense of motor competence reflect satisfied competence needs, shaped by supportive teacher feedback and individualised instruction (Vasconcellos et al., 2020). For students with SEN-ESD, PE may thus function as a rare context where recognising teacher interactions enable both need satisfaction and autonomous motivation. In contrast to the systematic recognition deficits for children with low motor skills in general PE (Gieß-Stüber & Grimminger-Seidensticker, 2023), for our students with SEN-ESD and described externalising EBPs, the profound misrecognition stems not from physical inability, but rather from being assessed on their social–emotional behaviour—precisely their area of need. For students with internalising EBPs, the performative demands of the gymnasium, including public exposure, may constitute a qualitatively different misrecognition risk.

Our *fourth main result* suggests that *misrecognition* in PE is not merely interpersonal but can also be understood as generated by a structural *grading paradox* revealed in *Pattern 5*, what we conceptualise as a double marginalization for students with SEN-ESD. To ground this interpretation in policy: the UN Convention on the Rights of Persons with Disabilities (United Nations, 2006), establishes an unconditional right to participation, and educational policies such as those of the German Standing Conference of the Ministers of Education and Cultural Affairs (KMK, 2011) mandate schools to ensure equitable access and to recognise and overcome barriers. In PE, this creates a particular tension: while curricula explicitly emphasise social-emotional skill development (e.g., DfE, 2013; KMK & DOSB, 2023; SHAPE America, 2024), students with and without SEN-ESD are subject to equivalent grading criteria for these competencies. The competitive, emotionally intense, and physically demanding nature of PE can be particularly overwhelming for these students given their challenges in perceiving, interpreting, and regulating emotions in social situations (Myschker & Stein, 2018). Interpreted through Prengel’s (2019) recognition theoretical framework and noting that this reading moves from student perceptions to theoretical implication, the institutional arrangement may culminate in what she would term an *adultist and ableist form of misrecognition*. The very social–emotional competencies that teachers rightly value as part of a Caring Community are simultaneously deployed as grading criteria within a standardised, hierarchizing system. This dynamic exemplifies what Dederich (2020) theorises as the ambivalence of recognition: institutionalised *criteria of recognisability*, such as standardised grading benchmarks risk recognising students *as* deficient, performing a subjection that fixes them in a limiting subject position (e.g., ‘the behaviourally challenged student’). Thus, the system designed to value social skills risks perpetuating the very symbolic degradation it is meant to overcome. As many curricula formally mandate the assessment of social–emotional competencies (e.g., KMK & DOSB, 2023; SHAPE America, 2024), students with SEN-ESD are thereby assessed on skills that are developmentally not yet consolidated (Myschker & Stein, 2018)—precisely those that constitute their formally diagnosed area of need. Our data show students perceive they are predominantly graded on “good behaviour,” “teamwork,” and “how we participate” (S7, Pos. 43; S8, Pos. 61). This even results in experienced misrecognition where some students report receiving lower grades specifically for behaviours related to their “handicaps” (S21, Pos. 130), while others perceive unfairness and confusion when peers with weaker motor skills receive comparable evaluations. Such grading practices as perceived by students may re-enact attachment trauma: teachers’ authority, which could provide protection, instead becomes a source of renewed symbolic violence (Brisch, 2018). Thus, the grading system becomes what Honneth identifies as a tool of symbolic degradation. To act with solidarity—in Honneth’s (1992/1995) and Prengel’s (2019) sense—would require valuing individual progress through individual reference norms and separating learning assessment from grading not yet consolidated competencies. As an implication, these findings suggest a need for *structural reform* beyond individual practice, such as individual reference norms (assessing progress relative to one’s own baseline), the separation of formative from summative assessment of social–emotional skills (whereby social–emotional skills remain a learning objective and are supported formatively, but do not enter summative grading), or legally defined compensation adjustments in PE for students with SEN-ESD. Without greater institutional alignment with inclusion principles, our data suggest that teachers’ efforts to foster interactional dignity could otherwise remain structurally constrained.

### 4.1. Strengths and Limitations

This study has several notable *strengths*. *First*, it addresses an *interdisciplinary empirical gap* as, to our knowledge, the first to qualitatively investigate recognition processes from the dual perspectives of students with SEN-ESD and their teachers. *Second*, the category of recognition emerged *inductively* from both participant groups, underscoring its importance. *Third*, *methodological rigor* was ensured through an iterative Grounded Theory process, where data collection and analysis proceeded until theoretical saturation (N = 40), with validation via interdisciplinary expert debriefing and practitioner feedback.

Two primary *limitations* should be considered. *First*, the findings are contextually grounded in German general schools with students holding formal SEN-ESD diagnoses. Additionally, externalising EBPs were confirmed through (special) educator report rather than standardised assessment; findings should therefore be interpreted cautiously as applying to students with educator-identified externalising profiles rather than psychometrically classified categories, and cannot be extended to internalising manifestations of SEN-ESD such as anxiety or social withdrawal. While this limits *generalizability*, the core findings regarding how recognising interactions are negotiated in teacher–student interactions to support inclusion may offer first insights for similar student groups in other educational systems. *Second*, the study’s design—*broad sampling* across groups rather than within specific teacher–student dyads—precludes analysis of mutual co-construction within relationships. Even dyadic interviews would, however, have produced reconstructions of each participant’s subjective PE reality rather than direct access to the interactional process itself.

### 4.2. Potential Future Directions

Building on this study’s model, future research should pursue four avenues. *First*, it could test whether the qualitative dimensions of interactional dignity translate to *quantitative measures* like the Being Seen Questionnaire to create a more accurate relational safety scale for students with and without SEN. This would refine the construct of teacher’s caring (Andresen et al., 2023) by differentiating items for proactive professional solidarity (e.g., structured check-ins) from those measuring severe boundary violations (e.g., public shaming). *Second*, research should test whether findings transfer to students with internalising EBPs, but also *beyond diagnostic labels* to all students with social–emotional needs as well as to *teacher–student dyads*. *Third*, research should explore approaches to addressing the *grading paradox*, which this study identifies as a potential source of double marginalization for students with SEN-ESD, though policy change is ultimately required to align assessment with inclusive principles. *Finally*, as recognition is the prerequisite for educational participation, research should trace how successfully negotiated recognition in PE concretely enables *inclusion* of students with SEN-ESD.

## 5. Conclusions

This study set out to understand how recognition is negotiated in interactions between teachers and students with Special Educational Needs in the area of Emotional and Social Development (SEN-ESD) in the inclusive Physical Education (PE) classroom. It reveals that interactional dignity is constituted through three core domains: relational security, fairness and voice, and the valuing of individual skills. Yet this process can be strained by perceptual discrepancies: one concerning what counts as fair (procedural equality versus needs-based treatment), and the other concerning whose reality is recognised. The first one (justice-related) leaves both groups in our data, teachers and students alike, perceiving the other’s fairness logic as unfair. The second (epistemic) occurs when students feel that teachers dismiss their subjective realities as merely subjective, thereby misrecognising their moral agency.

Consequently, this study makes two pivotal *contributions*. *Theoretically*, it addresses the critique that Honneth’s (1992/1995) model requires refinement for childhood contexts by integrating Prengel’s (2019) pedagogical specifications. This synthesised framework has proven valuable in reconstructing the specific recognition processes within inclusive PE for a vulnerable group; it adds Prengel’s empirically grounded analytical model to Honneth’s philosophical proposition. *Practically,* these findings point toward the need for a shift from individual teacher practice toward *institutional reform*. Beyond navigating the two perceptual discrepancies through dialogic engagement and transparent yet reversible rule practices, the identified *grading paradox* may function as a tool of *institutional misrecognition*: students with SEN-ESD risk being penalised for the very social–emotional competencies that define their area of need, due to the standardised assessment of these skills in many national PE curricula (e.g., KMK & DOSB, 2023; SHAPE America, 2024). Aligning policy with inclusion goals would therefore require structural reform, such as individual reference norms, the separation of formative from summative assessment, or legally defined compensation adjustments in PE for students with SEN-ESD. Without such alignment, teachers’ efforts to foster interactional dignity may remain structurally constrained, leaving students with SEN-ESD particularly vulnerable to double marginalisation when systemic and relational conditions are not addressed simultaneously.

The identified *limitations*, including the non-dyadic design and context-specific sample, precisely chart the course for *future research*. Essential next steps include dyadic studies to analyse the co-construction of these processes, and quantitative work to test and expand constructs such as the Being Seen Questionnaire (Andresen et al., 2023) with our dimensions of interactional dignity. Additionally, as recognition is the prerequisite for educational participation, research should investigate how successfully negotiated recognition is related to perceived inclusion in PE to support participation processes.

PE, through its unique curricular emphasis on social–emotional learning, presents a context of considerable dual potential for students with SEN-ESD: it may meaningfully support students’ social–emotional development and sense of belonging, or, if the conditions identified here remain unaddressed, it may compound the challenges they already face. Whether it leans toward one or the other depends substantially on the structural and relational conditions this study has sought to illuminate.

## Data Availability

Data cannot be shared publicly to protect participant privacy. The interview protocol, codebook and representative analytical memos are available from the corresponding author upon request.

## Note

1 We employ the term SEN-ESD (‘Förderschwerpunkt emotionale und soziale Entwicklung’) for its alignment with German legal frameworks (Conference of the Ministers of Education and Cultural Affairs guidelines). We acknowledge ongoing debates regarding its translation, where anglophone labels such as Emotional Behavioral Disorder (EBD) or Social Emotional Mental Health (SEMH) needs might be adopted.

## Abbreviations

The following abbreviations are used in this manuscript:

PE	Physical Education
SEN-ESD	Special Educational Needs in the area of Emotional and Social Development
SEN	Special Educational Needs
ESD	Emotional and Social Development
DfE	Department for Education
KMK	Kultusministerkonferenz
EBP	Emotional and Behavioural Problems
SDT	Self-Determination Theory

## References

[B1-behavsci-16-00793] Achenbach T. M., Ivanova M. Y., Rescorla L. A., Turner L. V., Althoff R. R. (2016). Internalising/externalising problems: Review and recommendations for clinical and research applications. Journal of the American Academy of Child and Adolescent Psychiatry.

[B2-behavsci-16-00793] American Psychiatric Association (2013). Diagnostic and statistical manual of mental disorders: Dsm-5.

[B3-behavsci-16-00793] Andresen F., Torvik E., Lagestad P. (2023). Students’ experience of being seen by their physical education teachers and associated factors. Frontiers in Sports and Active Living.

[B4-behavsci-16-00793] Boger M.-A., Mittertrainer M., Oldemeier K., Thiessen B. (2023). Theorien der inklusion—Eine übersicht [Theories of inclusion—A review]. Diversität und diskriminierung: Analysen und konzepte.

[B5-behavsci-16-00793] Breuer F., Muckel P., Dieris B. (2018). Reflexive grounded theory: Eine einführung für die forschungspraxis *[Reflexive grounded theory: An introduction for research practice]*.

[B6-behavsci-16-00793] Brisch K. H., Brisch K. H. (2018). Trauma ist nicht gleich trauma: Die spezifischen auswirkungen von bindungstraumatisierungen auf opfer, täter und behandler [Trauma is not all the same: The specific effects of attachment-related traumatization on victims, perpetrators, and therapists]. Bindungstraumatisierungen: Wenn bindungspersonen zu tätern werden.

[B7-behavsci-16-00793] Brunssen L., Haase R. K., Kastrup V. (2025). Encouraging inclusion of students with social, emotional, and mental health needs in physical education: A systematic review. German Journal of Exercise and Sport Research.

[B8-behavsci-16-00793] Brunssen L., Kastrup V. (2025). Teacher sensitivity as a bridge to emotion regulation for students with special educational needs in their emotional and social development in physical education. Frontiers in Sports and Active Living.

[B9-behavsci-16-00793] Corbin J. M., Strauss A. L. (2008). Basics of qualitative research: Techniques and procedures for developing grounded theory.

[B10-behavsci-16-00793] Dederich M. (2020). Anerkennung und vulnerabilität: Inklusionspädagogische überlegungen in anschluss an butler und levinas [Recognition and vulnerability: Inclusive education reflections following butler and levinas]. Zeitschrift für Inklusion.

[B11-behavsci-16-00793] Department for Education (2013). The national curriculum in England: Key stages 1 and 2 framework document.

[B12-behavsci-16-00793] Gieß-Stüber P., Grimminger-Seidensticker E., Gieß-Stüber P., Tausch B. (2023). Abwertung und ausgrenzung vermeiden: Pädagogische und didaktische überlegungen zur anerkennungsförderlichen gestaltung von sportangeboten im kindes- und jugendalter [Avoiding devaluation and exclusion: Pedagogical and didactic considerations for designing recognition-promoting sports programmes in childhood and adolescence]. Gesellschaftlicher zusammenhalt im und durch sport.

[B13-behavsci-16-00793] Grimminger E. (2012). Anerkennungs- und missachtungsprozesse im sportunterricht: Die bedeutung von machtquellen für die gestaltung sozialer peer-beziehungen [Processes of recognition and non-recognition in physical education: The meaning of power sources for the construction of peer relations]. Sportwissenschaft.

[B14-behavsci-16-00793] Guthöhrlein K., Laubenstein D., Scheer D. (2025). Präventive unterstützung von kompetenzen der emotionalen und sozialen entwicklung durch wertschätzende und lösungsfokussierte kommunikationsstrategien: Dem guten grund auf der spur [Preventive support of emotional and social development competencies through appreciative and solution-focused communication strategies: Tracing the ‘good reason’]. Zeitschrift für Heilpädagogik.

[B15-behavsci-16-00793] Honneth A., Anderson J. (1995). The struggle for recognition: The moral grammar of social conflicts.

[B16-behavsci-16-00793] Julius H., Gasteiger-Klicpera B., Kißgen R. (2009). Bindung im kindesalter: Diagnostik und interventionen *[Attachment in childhood: Assessment and interventions]*.

[B17-behavsci-16-00793] Kultusministerkonferenz (2011). Inklusive bildung von kindern und jugendlichen mit behinderungen in schulen: Beschluss der kultusministerkonferenz vom 20.10.2011 *[Inclusive education of children and adolescents with disabilities in schools: Resolution of the standing conference of the ministers of education and cultural affairs of 20 October 2011]*.

[B18-behavsci-16-00793] Kultusministerkonferenz, Deutscher Olympischer Sportbund (2023). Gemeinsame handlungsempfehlungen der kultusministerkonferenz und des Deutschen Olympischen sportbundes zur weiterentwicklung des schulsports 2023 bis 2028: Beschluss der kultusministerkonferenz vom 10.11.2023 *[Joint recommendations of the standing conference of the ministers of education and cultural affairs and the German Olympic sports confederation for the further development of school sports 2023–2028: Resolution of 10 November 2023]*.

[B19-behavsci-16-00793] Medcalf R., Marshall J., Hardman K., Visser J. (2011). Experiences and perceptions of physical education. Emotional and Behavioural Difficulties.

[B20-behavsci-16-00793] Müller T. (2017). “Ich kann niemandem mehr vertrauen”: Konzepte von vertrauen und ihre relevanz für die pädagogik bei verhaltensstörungen *[“I can no longer trust anyone”: Concepts of trust and their relevance for pedagogy in behavioral disorders]*.

[B21-behavsci-16-00793] Myschker N., Stein R. A. (2018). Verhaltensstörungen bei kindern und jugendlichen: Erscheinungsformen, ursachen, hilfreiche massnahmen *[Behavioral disorders in children and adolescents: Manifestations, causes, and helpful interventions]*.

[B22-behavsci-16-00793] Opp G. (2017). Schmerzbasiertes verhalten: Eine paradoxe pädagogische herausforderung [Pain-based behaviour: A paradoxical pedagogical challenge]. Zeitschrift für Heilpädagogik.

[B23-behavsci-16-00793] Popp K., Methner A., Zimmermann D., Meyer M., Hoyer J. (2016). Inklusive beschulung von kindern und jugendlichen im förderschwerpunkt—Nicht nur eine herausforderung an das schulsystem [Inclusive schooling of children and adolescents in special educational needs contexts—Not just a challenge for the school system]. Ausgrenzung und teilhabe: Perspektiven einer kritischen sonderpädagogik auf emotionale und soziale entwicklung.

[B24-behavsci-16-00793] Prengel A., Lutz H., Wenning N. (2001). Egalitäre differenz in der bildung [Egalitarian difference in education]. Unterschiedlich verschieden.

[B25-behavsci-16-00793] Prengel A. (2019). Pädagogische beziehungen zwischen anerkennung, verletzung und ambivalenz *[Pedagogical relationships between recognition, injury, and ambivalence]*.

[B26-behavsci-16-00793] Robert Koch-Institut (2018). KiGGS welle 2—Gesundheitliche lage von kindern und jugendlichen [KiGGS wave 2—Health status of children and adolescents]. Journal of Health Monitoring.

[B27-behavsci-16-00793] Ryan R. M., Deci E. L. (2017). Self-determination theory: Basic psychological needs in motivation, development, and wellness.

[B28-behavsci-16-00793] SHAPE America (2024). National physical education standards.

[B29-behavsci-16-00793] Thomas N. P. (2012). Love, rights and solidarity: Studying children’s participation using Honneth’s theory of recognition. Childhood.

[B30-behavsci-16-00793] Thomas N. P., Martin C., Diter K. (2024). Well-being, recognition and participation: The challenge for schools. Well-being at school: A social problem.

[B31-behavsci-16-00793] United Nations (2006). Convention on the rights of persons with disabilities.

[B32-behavsci-16-00793] Vasconcellos D., Parker P. D., Hilland T., Cinelli R., Owen K. B., Kapsal N., Lee J., Antczak D., Ntoumanis N., Ryan R. M., Lonsdale C. (2020). Self-determination theory applied to physical education: A systematic review and meta-analysis. Journal of Educational Psychology.

[B33-behavsci-16-00793] Woodgate R. L., Gonzalez M., Demczuk L., Snow W. M., Barriage S., Kirk S. (2020). How do peers promote social inclusion of children with disabilities? A mixed-methods systematic review. Disability and Rehabilitation.

[B34-behavsci-16-00793] World Health Organization (2022). ICD-11: International classification of diseases *(11th revision)*.

[B35-behavsci-16-00793] Zimmermann D. (2025). Psychosoziale beeinträchtigungen bei kindern und jugendlichen: Erkennen, verstehen, beziehung gestalten *[Psychosocial impairments in children and adolescents: Recognizing, understanding, and shaping relationships]*.

